# A New Stochastic Technique for Painlevé Equation-I Using Neural Network Optimized with Swarm Intelligence

**DOI:** 10.1155/2012/721867

**Published:** 2012-07-11

**Authors:** Muhammad Asif Zahoor Raja, Junaid Ali Khan, Siraj-ul-Islam Ahmad, Ijaz Mansoor Qureshi

**Affiliations:** ^1^Department of Electronic Engineering, International Islamic University, Islamabad, Pakistan; ^2^Center for Computational Intelligence, P.O. Box 2300, Islamabad, Pakistan; ^3^Department of Electrical Engineering, COMSATS Institute of Information Technology, Attock, Pakistan; ^4^Pakistan Institute of Engineering and Applied Science, Nilore, Pakistan

## Abstract

A methodology for solution of Painlevé equation-I is presented using computational intelligence technique based on neural networks and particle swarm optimization hybridized with active set algorithm. The mathematical model of the equation is developed with the help of linear combination of feed-forward artificial neural networks that define the unsupervised error of the model. This error is minimized subject to the availability of appropriate weights of the networks. The learning of the weights is carried out using particle swarm optimization algorithm used as a tool for viable global search method, hybridized with active set algorithm for rapid local convergence. The accuracy, convergence rate, and computational complexity of the scheme are analyzed based on large number of independents runs and their comprehensive statistical analysis. The comparative studies of the results obtained are made with MATHEMATICA solutions, as well as, with variational iteration method and homotopy perturbation method.

## 1. Introduction

The history of Painlevé equations is more than one century old. These are six second-order nonlinear irreducible equations that define new transcendental functions known as Painlevé Transcendents. These functions describe different physical processes and have been extensively used in both pure [1] and applied mathematics [2], along with theoretical physics [3]. For instance, Painlevé Transcendents are used in solutions to Korteweg-de Vries (KdV), cylindrical KdV and Bussinesq equations [4, 5], bifurcations in nonlinear non-integral models [6], matrix models of quantum gravity with continuous limits [7, 8], among others. The history, importance, and applications of Painlevé transcendents can be seen elsewhere [9, 10]. In recent publications, Painlevé equation-I (PE-I) is used in Tronquée [11] and hyperasymptotic [12] solutions, as well as in modeling the viscous shocks in Hele-shaw flow and Stokes phenomena [13]. Beside this, many researchers have employed PE-I in diverse fields of applied science and engineering [14–22].

In this article, our investigation is confined to find out solution of initial value problem (IVP) of nonlinear second-order PE-I, written in the following form:

(1) y′′(t)=6y(t)2+t, t∈(0,1),y(0)=0,  y′(0)=1.

In the present study, the strength of feed forward artificial neural networks (ANNs) is exploited for approximate mathematical model of PE-I. The real strength of such model to solve the differential equations can be achieved by using modern stochastic solvers for optimization of weights based on particle swarm optimization (PSO) technique hybrid with local search methods. For example, the solution of linear and nonlinear differential equations and their systems is reported extensively by many researchers [23–26]. Recently, solution of well-known fractional-order systems in engineering based on Bagley-Torvik and Riccati equations are other illustrative examples of such approaches [27, 28].

In this paper, ANNs supported with PSO and active set algorithm (ASA) are used to find the solution of IVP of PE-I. Monte-Carlo simulations of proposed scheme are performed and analyzed statistically to verify and validate its effectiveness. Comparison with standard MATHEMATICA, as well as homotopy perturbation method (HPM) [29, 30] and variational iteration method (VIM) [29, 30] results is also carried out for the given scheme.

The organization of the paper is as follows: the objective function creation with neural network mathematical modeling and its training methodology are introduced in Section 2. A brief introduction to particle swarm optimization algorithm is revealed in Section 3. A detailed application of the designed method along with some discussion on the results is presented in Section 4. Comparative analysis of the results is presented in Section 5. Last section concludes the findings along with some directions for future research.

## 2. Mathematical Modeling

The Feed-forward ANNs is well known to be used as a universal function approximator for any continuous function along with its derivatives on a compact set. In the modeling of differential equation through ANN methodology, following continuous mapping is used for the solution *y*(*t*), first-order derivative *y*′(*t*) and second-order derivative *y*′′(*t*), respectively, given as [31–34]

(2) y^(t)=∑i=1mαif(wit+βi),y^′(t)=∑i=1mαif′(wit+βi),y^′′(t)=∑i=1mαif′′(wit+βi),

where *α*_*i*_, *w*_*i*_, and *β*_*i*_ are real-valued bounded adaptive parameters, *m* is the number of neurons, and *f* being the activation function taken as log sigmoid for hidden layers:

(3) f(x)=11+e−x.

**Figure 1:**
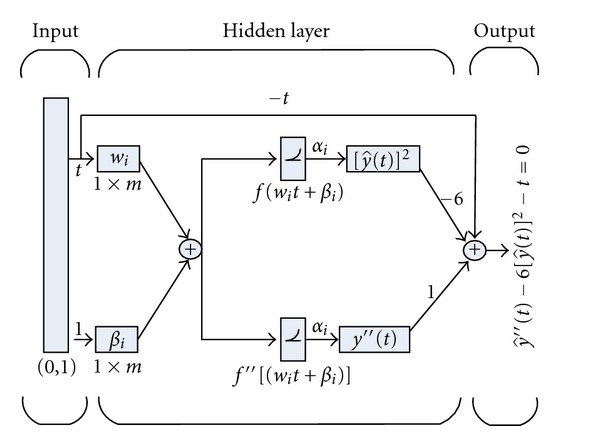
DE-NN architecture for Painlevé equation I.

The linear combination of the networks represented by (2) can arbitrarily model the nonlinear PE-I. It is called differential equation neural network (DE-NN) of the equation and its architecture is shown in Figure 1.

The objective or fitness function *e* for solving PE-I has been formulated by defining an unsupervised error, given as the sum of mean square errors:

(4) e=e1+e2|j, j=1,2,3,…,M,

where *j* is the iteration number, *M* represents the total number of iterations, and the error term *e*_1_ is related with the differential equation and is written as

(5) e1=1N+1∑m=0N(y^m′′−6y^m2−tm)2, t∈(0,1),N=1h,  y^m=y^(tm), tm=mh.

Here, the interval *t* ∈ (0, 1) is divided into *N* number of steps *t* ∈ (*t*_0_ = 0, *t*_1_, *t*_2_,…, *t*_*N*_ = 1) with step size *h* and $\hat{y}(t)$ and ${\hat{y}}^{\prime \prime }(t)$ are DE-NN given in set of (2).

Similarly, the error term *e*_2_ is due to initial conditions and it is given as

(6) e2=12(y^02+(y^0′−1)2).

It is quite clear that, with the availability of weights *α*_*i*_, *w*_*i*_, and *β*_*i*_ in DE-NN model for which the values of functions *e*_1_ and *e*_2_ approach zero, the value of unsupervised error *e* also approaches zero. Therefore, the solution *y*(*t*) of the PE-I is approximated by $\hat{y}(t)$ as given in (2).

## 3. Learning Methodology 

The learning procedures are introduced here for finding the unknown weights of DE-NN networks representing PE-I using PSO and ASA algorithms.

The swarm intelligence techniques often referred to as PSO algorithm was first introduced by Kennedy and Eberhart [35]. It is a kind of global optimization technique which was based on the model of social behavior of bird flocking and fish schooling. Its discrete and continuous versions have been developed and used in different optimization problems of applied science and engineering. Few examples include the application to mobile communications, sensor networks, inventory control, multiprocessor scheduling, controls, stock market prediction, and steganographic methods [36–39] and so forth.

In PSO algorithm, each single candidate solution to an optimization problem is taken as a particle in the search space. The exploration of a problem space in PSO algorithm is made with a population of particles called a swarm. All particles in the swarm have own fitness values associated with problem specific objective function. The PSO algorithm is initialized with a swarm of particles randomly and is used to search for optimal solution iteratively. The broader spread of initial swarm results in optimal performance of the algorithm. The position and the velocity of each particle are updated according to its known previous local best position **P**_*lb*_^*n*−1^ and the global best position of all particles **P**_*gb*_^*n*−1^ in the swarm so far. The updating formula for each particle velocity and position in continuous standard PSO is written as

(7) vin=ωvin−1+c1r1(Plbn−1−xin−1)+c2r2(Pgbn−1−xin−1)xin=xin−1+vin−1,

where **x**_*i*_ is vector to represent *i*th particle of the swarm, *i* = 1, 2, …, *p*,  *p* is an integer giving the total number of particles in a swarm. The **v**_*i*_ is the velocity vector associated with *i*th particle, *c*_1_ and *c*_2_ are the local and global social acceleration constants, respectively, *ω* is the inertia weight linearly decreasing over the course of search between 0 and 1, and **r**_1_ and **r**_2_ are random vectors with elements distributed between 0 and 1.

**Figure 2:**
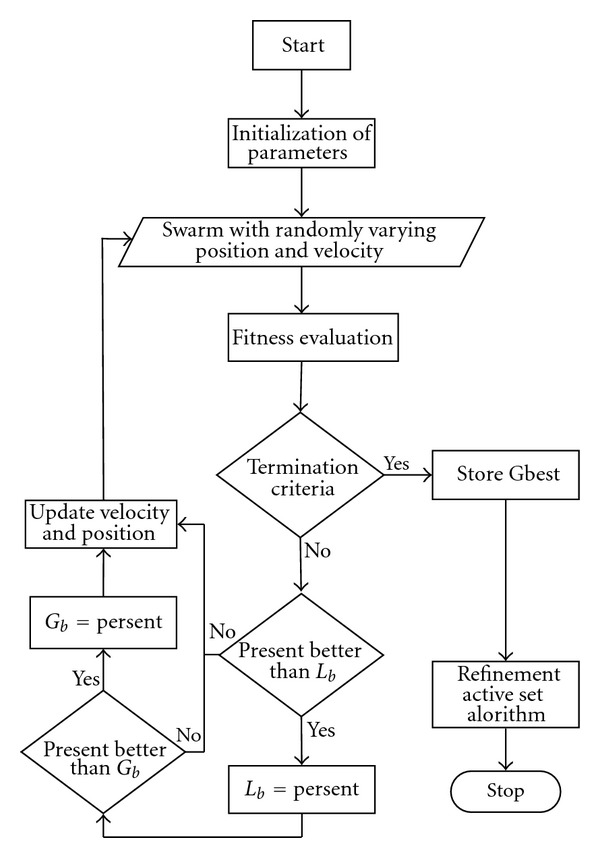
Flowchart of particle swarm optimization aided with actives set algorithm.

The elements of velocity are bounded as *v*_*i*_ ∈ [−*v*_max⁡_, *v*_max⁡_], where *v*_max⁡_ is maximum velocity. If the velocity goes beyond its maximum value, it will be set to *v*_max⁡_. This parameter has its own importance and controls the convergence rate of the algorithm. The execution of the algorithm is terminated on the criterion to maximum flights/cycles is completed or a specific value of the fitness is achieved. The generic flowchart showing the process of proposed algorithm is presented in Figure 2.

The algorithm is given in the following steps.

**Step 1**

*Initialization.* The initial swarm of particle is generated randomly using bounded real values. Each particle represents the candidate solution with as many members as number of unknown parameters in DE-NN networks. The scattering in values of initial swarm is maintained for better search space for the algorithm. Initialize the values of parameters as given in Table 1 before execution of algorithm.

**Step 2**

*Fitness Evaluation.* Calculate fitness for each particle by using the fitness function given in (4), (5), and (6).

**Step 3**

*Termination Criteria.* Terminate the algorithm if any of following criteria satisfies the following:

- predefined value of fitness achieved that is, *e* ≤ 10^−10^,
- number of maximum flights/cycles is executed.

If yes, then go to Step 6.

**Step 4**

*Ranking.* Rank each particle on the basis of minimum of the fitness function values.

**Step 5**

*Renewal.* Update the velocity and position are updated using the equations given in (7). Repeat the algorithm from Step 2 to Step 5 until total number of flights is reached.

**Step 6**

*Refinement.* MATLAB built-in formulation is used for running active set algorithm (ASA) for fine tuning of the results. The global best particle of PSO is used as a start point of the algorithm and other parameter settings for ASA is also provided in Table 1.

**Step 7**

*Statistical Analysis.* Store the value of global best particle along with its fitness value and time of executions for algorithm. Repeat Step 1 to 6 for sufficient large number of runs for reliable statistical analysis.

## 4. Simulation and Results

The well-known analytic solvers like adomian decomposition method (ADM), variational iteration method (VIM), homotopy perturbation method (HPM), homotopy analysis method (HAM), and their modified versions are used extensively to provide the solutions of strong nonlinear Painlevé equations [29, 30, 40–42]. The generic solution is provided in all these mentioned methods in the form of infinite series, but the results are determined using finite number of terms, whose accuracy can be enhanced with the increase in number of terms.

Before describing the designed approach, a brief description of VIM and HPM has been provided for PE-I. The approximate solution of *y*(*t*) for PE-I (1) in case of both VIM and HPM is represented in the form of a function *u*(*t*). To describe the VIM, consider the following nonlinear differential equation:

(8) L(u)+N(u)=g(t),

where *L* and *N* are linear and nonlinear operators, respectively, and *g*(*t*) is an inhomogeneous term. In standard VIM, the correct functional can be constructed as

(9) un+1(t)=un(t)+∫0tλ(τ)(Lun(t)+Nu~n(τ)−g(τ))dτ,

here, *λ*(*τ*) represents a general Lagrangian multiplier, *n* and *n* + 1 are the present and next approximation, ũ represents restricted variation, that is, *δũ* = 0. With suitable choice of *u*_0_, the fixed point of the correction functional (9) can be considered as an approximate solution of the equation and successive approximation *u*_*n*+1_ of the solution *u* will be readily obtained upon by determining the Lagrange multiplier. Consequently, the solution is given by *u* = lim⁡_*n*→*∞*_⁡*u*_*n*_. For interesting readers, the detail description of VIM along with its applicability to various kinds of differential equations can be seen in [43–46].

The governing iterative formulation for solving the PE-I using VIM with the optimal value for *λ*(*τ*) = *τ* − *t* can be written as

(10) un+1(t)=un(t)+∫0t(τ−t)(un′′(τ)−6u~n2(τ)−τ)dτ,

The approximate solution *u*(*t*) by VIM for first three iterations using *u*_0_(*t*) = *t* + (1/6)*t* ^3^ can be written as

(11) u1(t)=t+16t3+12t4+115t6+1336t8,u2(t)=t+16t3+12t4+115t6+17t7+⋯+187764400t14+1100800t16+15757696t18,u3(t)=t+16t3+12t4+115t6+17t7+⋯+11871210325276160000t14+160939454466400t36+17768399149858816t38.

On the other hand to explain the HPM, let us consider the following general equation of type:

(12) A(u)−g(t)=0, t∈Ω,

where *A* represents the differential operator, *Ω* is the domain and *g*(*t*) is a known analytical function. The operator *A* can consist of linear part *L* and a nonlinear part *N*. In standard HPM, the Hopotopy *v*(*r*, *p*) : *Ω* × [0, 1] → *R* can be constructed which satisfies

(13) H(v,p)=(1−p)(L(v)−L(u0))+p(A(v)−g(t))=0,

here, *p* ∈ [0, 1] is an embedding parameter and *u*_0_ is an initial approximation of (12). By using small embedding parameter *p*, the *v*th solution of (12) can be given as power series in *p*, that is,

(14) v=v0+pv1+p2v2+⋯,

for *p* = 1, the approximate solution for (12) is given as

(15) u=lim⁡p→1v=v0+v1+v2+⋯.

The method provides enough convergence in most of the cases and the rate of convergence depends on the nonlinear operator *A*(*v*). The detail description of the HPM and its applications can be seen in [47–50].

Using HPM, the solution of PE-I (1) starting from *u*_0_(*t*) = *t* + (1/6)*t* ^3^ can be given as

(16) u=u(0)+tu′(0)+16t3+6∬0tu2dt dt.

using the initial conditions (1), the homotopy constructed by the method can be given as

(17) H(u,p)=u−t−16t3+6p∬0tu2dt dt=0.

Inserting ∑_*i*=0_^*∞*^ *p* ^*i*^ *u*_*i*_ instead of *u* and comparing the coefficients of 1, 2, 3, 4, and 5 powers of *p*, the approximate solutions by HPM for PE-I are, respectively, given as

(18) u1(t)=t+16t3+12t4+115t6+1336t8,u2(t)=t+16t3+12t4+115t6+17t7+⋯+140t9+1746200t11+126208t13,u3(t)=t+16t3+12t4+115t6+17t7+⋯+52198408400t14+3551144144000t16+95224550144t18,u4(t)=t+16t3+12t4+115t6+17t7+⋯+16346914378364000t19+163451491203440000t21+14580637139501513648000000t23,u5(t)=t+16t3+12t4+115t6+17t7+⋯+404709982723087434930560000t24+14580637139501513648000000t26+748491737058836344832t28.

Along with the analytical solutions given by VIM and HPM, the results are also calculated with MATHEMATICA *y*_*M*_(*t*). These results are used for comparison with the solution provided by the proposed design scheme.

Mathematical model of the equation is made with DE-NN networks given by (2) by taking 10 numbers of neurons, resulting in 30 unknown weights in DE-NN networks. The weights are taken as bounded real numbers between −50 to 50. The initial swarm consists of 160 particles, each with 30 numbers of elements, which is equivalent to the number of weights for DE-NN networks. Input of the training set is taken as *t* ∈ (0,1) with a step of *h* = 0.1. The fitness function provided in (4) can be formulated as

(19) e=111∑m=010(y^m′′−6y^m2−tm)2+12(y^02+(y^0′−1)2)|j,             j=1,2,3,…,2000.

The PSO algorithm runs iteratively in order to compute the minimum of fitness function (19) and the best particle of PSO technique is passed to ASA algorithm as a start point for rapid local search. The parameter settings for the execution of algorithms are provided in Table 1.

Similarly, hundred independent runs of PSO and PSO hybrid with ASA (PSO-ASA) algorithms have been performed for finding the optimal weights. The weights obtained by the algorithms are used in (2) to obtained the proposed solutions $\hat{y}(x)$ of PE-I. One set of weights for which the value of fitness function 1.3699 × 10^−04^ and 9.1210 × 10^−08^ for PSO and PSO-ASA, respectively, are provided in Table 2. Results calculated by these weights are provided in Tables 3 and 4 for inputs between 0 and 1 with a step of 0.1. The solutions of 3rd order VIM, 5th order HPM for the same inputs are also given in Table 3 while detailed information has been provided in Table 4. It can be seen that the value of mean absolute error (MAE) for 3rd order VIM, 5th order HPM, PSO, and PSO-ASA techniques are 4.6098 × 10^−04^, 7.6946 × 10^−05^, 9.8594 × 10^−04^, 1.1198 × 10^−05^, respectively.

The values of minimum (MIN), maximum (MAX), mean, and standard deviation (STD), for absolute error (AE) $\left|y_{M}(t)-\hat{y}(t)\right|$, are calculated on the basis hundred independent runs for PSO and PSO-ASA methods. Results are provided in the Table 5 for inputs 0 and 1 with step of 0.1 for PE-I. It can be seen that values of AE on the basis of MIN are of the order 10^−03^ to 10^−04^ and 10^−06^ to 10^−08^ for PSO and PSO-ASA algorithms, while the values of AE based on mean lies in the range 10^−01^ to 10^−02^ and 10^−04^ to 10^−05^, respectively, for said solvers. The lowest values of statistical parameters are found by hybrid technique PSO-ASA than that of PSO algorithm.

**Figure 3:**
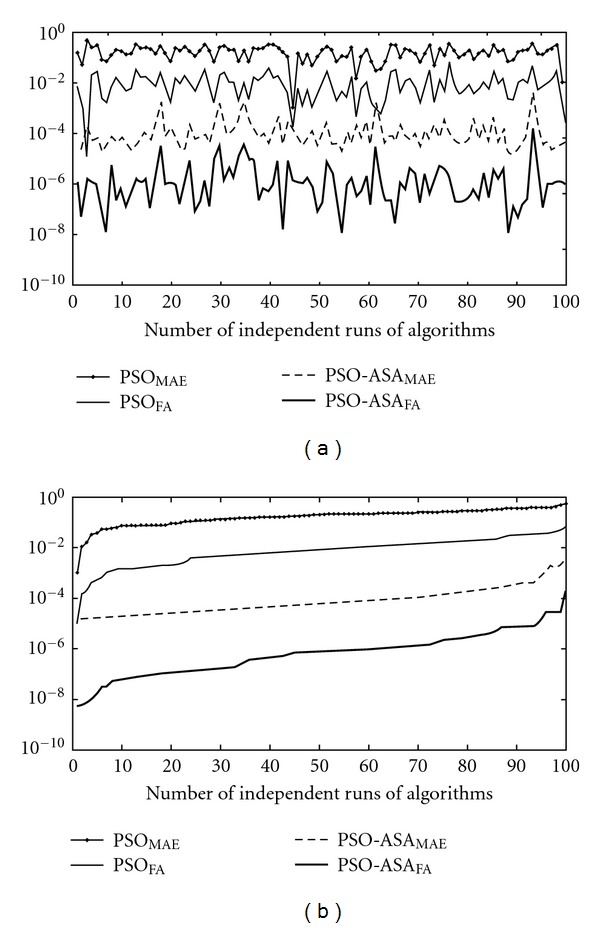
Comparison of results on the basis of fitness achieved (FA) and mean absolute error (MAE).

The accuracy and convergence of the algorithms are analyzed further by calculating the values of MAE and fitness achieved (FA) for 100 independents runs. Results are shown graphically on semilog scale in order to elaborate the difference in the values. The value of FA and MAE against number of independent runs are shown graphically in Figure 3(a) for PSO and PSO-ASA methods. The numbers of independent runs are arranged on the basis of ascending order of the fitness achieved, as well as values of MAE and are plotted in Figure 3(b). On the basis of acceptability criteria of MAE ≤ 10^−03^, the convergence for PSO and PSO-ASA is 1% and 100%, respectively.

## 5. Comparative Analysis of Results

In this section, the comparative study for results is presented based on values of MAE, execution time (ET), mean fitness achieved (MFA), global mean absolute error (GMAE), number of grid points, and convergence rate for PSO and PSO-ASA proposed solvers.

The effects on results by changing the number of gird points used in fitness function (19) are analyzed. By taking the number of grid points 21 instead of 11, the new fitness function is formulated using the input of the training set *t* ∈ (0,1) with a step of *h* = 0.05 and given as

(20) e=121∑m=020(y^m′′−6y^m2−tm)2+12(y^02+(y^0′−1)2)|j,                j=1,2,3,…,2000.

Hundred independents runs of PSO and PSO-ASA method are executed for finding the appropriate weights and subsequently the solution of IVP of PE-1. The MFA is defined as average values of fitness achieved taken over all independent runs of the algorithm. Similarly, GMAE is average value of mean absolute error for all independents runs of solvers. The values of MFA and GMAE are calculated for PSO and PSO-ASA techniques using both 11 and 21 grids points and results are tabulated in Table 6. It is found that the values of MFA are of the order 10^−02^ and 10^−06^ for PSO and PSO-ASA, respectively. The values of MAE are matched with MATHEMATICA solution *y*_*M*_(*t*) upto 2 to 3 and 5 to 8 decimal places for PSO and PSO-ASA techniques. The value of GMAE for 11 and 21 grids points are determined and given in Table 6. It is found that values of GMAE are of the order 10^−04^ for both the cases for inputs between 0 and 1 with a step of *h* = 0.1. So, by increasing number of grid points the same level of the accuracy in results is maintained for both PSO and PSO-ASA techniques. The increase in number of grids points, that is, decrease in the mesh size *h*, does not guaranty improvement of results for stochastic solvers and same is observed in our simulations by changing the value of step size *h* = 0.1 to *h* = 0.05.

The convergence rates of the proposed stochastic solvers are determined based on the value of MAE ≤10^−02^, 10^−03^, and 10^−04^, as well as the value of MFA *e* ≤ 10^−03^, 10^−04^, and 10^−05^. Results are tabulated in Table 6 for both PSO and PSO-ASA techniques. It can be seen that, with the acceptability of the results based on value of MFA *e* ≤ 10^−05^, the hybrid approach PSO-ASA achieved this criteria for 99% and 98% of the independents runs for 11 and 21 numbers of grid points, respectively. In addition to that, results are consistently convergent for larger number of independents runs for PSO-ASA method than that of PSO algorithms.

Moreover, the computational complexity is analyzed by calculating the time of execution of each algorithm for 100 numbers of independent runs for both *h* = 0.1 and *h* = 0.05. The values for minimum execution time (MIN-ET), maximum execution time (MAX-ET), mean execution time (M-ET), and standard deviation of execution time (STD-ET) are provided in the Table 6. It can be seen that values of M-ET for PSO and PSO-ASA techniques are 76.93 seconds and 92.17 seconds, respectively, for *h* = 0.1, and almost double values of M-ET in case for *h* = 0.05. The time taken for the execution for hybrid approach PSO-ASA is relatively greater than that of PSO algorithm, but this can be overshadow by its invariable dominance in accuracy and convergence. The time analysis provided in this article is carried out using Dell Latitude D630 laptop with Intel(R) Core(TM) 2 Duo CPU T9300, 2.50 GHz, and running with MATLAB version R2009b.

## 6. Conclusion

On the basis of the simulations and their comparative studies provided in the last sections, it can be concluded that the IVP of PE-I can be solved effectively by the designed computational intelligence algorithms using neural networks supported with PSO and ASA techniques. Results obtained using the weights for DE-NN networks of PE-I trained by PSO-ASA algorithm are found to be better than from PSO techniques. The values of absolute error from MATHEMATICA solution *y*_*M*_(*t*) of PSO-ASA lies in the range 10^−04^ to 10^−07^ and match upto 5 to 7 decimal places with state of art analytical techniques of VIM and HPM. 

On the basis of 100 independent runs for each algorithm to solve PE-I using 11 and 21 grid points, the PSO-ASA technique is found to be invariable superior than PSO algorithm using the criteria based on the values of mean absolute error, global mean absolute error, mean fitness achieved, and convergence rate. However, as far as computational complexity is concerned that PSO-ASA technique is slightly more computational exhaustive, but it can be overlook due to its overall dominance in the performance. This study can also be extended to use other biological inspired computational algorithms for solving the PE-I, as well as other Painlevé transcendents.

## Figures and Tables

**Table 1:** Parameter settings for ASA and PSO algorithms.

PSO		ASA	
Parameters	Setting	Parameters	Setting
Swarm Size	160	Start Point	Best Particle of PSO
Particle size	30	No. of variable	30
Flights	2000	Iteration	1000
*c*_1_	Linear decreasing (2.5 to 0.5)	Maximum function Evaluations (MaxFunEvals)	50000
*c*_2_	Linear increasing (0.5 to 2.5)	Function tolerance (TolFun)	10^-18^
*ω*	Linearly decreasing (0.9 to 0.4)	Nonlinear Constraints tolerance (TolCon)	10^-18^
*v*_max⁡_	02	Derivative approximate	Finite forward difference
Population Span	(−50, 50)	X-Tolerance (TolX)	10^-12^
Velocity Span	(−2, 2)	Bounds	(−50, 50)

**Table 2:** A set of weights trained for DE-NN networks.

	PSO	PSO-ASA		PSO	PSO-ASA
*w*_1_	11.003223968159700	−7.607757117408950	*w*_6_	2.212628719834520	2.474582479870870
*w*_2_	1.215553756976230	−1.490826886546450	*w*_7_	−3.435984020017740	1.576108544205200
*w*_3_	−0.105724097992031	−0.545895962495148	*w*_8_	3.162425319231580	−3.958973407625250
*w*_4_	−0.631338164295177	7.264468183878910	*w*_9_	0.783489331068287	1.998216516692480
*w*_5_	5.142485647745030	−8.830221260895670	*w*_10_	−1.369123625736370	0.021432666153674

*α*_1_	2.212942902261790	1.227208825263930	*α*_6_	1.400872158822050	1.316114527930020
*α*_2_	8.952236157349910	−3.632183934646160	*α*_7_	−0.420235540017917	1.323004693243890
*α*_3_	0.211094325564814	4.680032352849500	*α*_8_	0.771318701716261	−2.560496480052050
*α*_4_	−0.918096490077305	9.995894668412700	*α*_9_	−1.394533732778610	1.004134898951920
*α*_5_	34.739272824112700	−0.083663885403684	*α*_10_	−0.343093197542097	0.887019181878860

*β*_1_	−14.756692394364600	8.897738213117890	*β*_6_	1.201391820838980	1.100068363593600
*β*_2_	−3.441636802273880	2.675860058162530	*β*_7_	3.935692917406700	0.587872446048014
*β*_3_	2.708026281856620	−0.220415897788109	*β*_8_	−3.847728945588120	5.075428578185270
*β*_4_	1.986093010073140	−9.999999632285810	*β*_9_	−1.648848649331190	−2.429219273541780
*β*_5_	−9.056219460761080	8.881048710700930	*β*_10_	−0.531535793135294	2.174973763274050

**Table 3:** Comparison of the results for solution of Painlevé I.

*T*	*y*_*M*_(*t*)	Results for *u*(*t*)		Proposed Results $\hat{y}(t)$	
		VIM	HPM	PSO	PSO-ASA
0.1	0.1002167469	0.1002167477	0.1002167477	0.101267314	0.100211733
0.2	0.2021394539	0.2021394527	0.2021394527	0.202944631	0.202137722
0.3	0.3086307548	0.3086307489	0.3086307492	0.309301843	0.308629917
0.4	0.4239862999	0.4239862788	0.4239862895	0.424625376	0.423985302
0.5	0.5543401416	0.5543399112	0.5543401182	0.555018673	0.554341815
0.6	0.7084621313	0.7084596600	0.7084620603	0.709234588	0.708467300
0.7	0.8992500131	0.8992296942	0.8992493803	0.900159983	0.899254787
0.8	1.1465318491	1.1463982509	1.1465240263	1.147598102	1.146541253
0.9	1.4825246345	1.4817789520	1.4824439956	1.483815424	1.482531252
1.0	1.9631285609	1.9594210423	1.9624483064	1.965104083	1.963204324

**Table 4:** Comparison of the results for solution of Painlevé I.

*T*	*u*_*k*_(*t*) for VIM			*u*_*k*_(*x*) for HPM				DE-NN	
	*k* = 1	*k* = 2	*k* = 3	*k* = 2	*k* = 3	*k* = 4	*k* = 5	PSO	PSO-ASA
0.1	1.35*E* − 08	8.00*E* − 10	8.00*E* − 10	7.96*E* − 10	8.00*E* − 10	8.00*E* − 10	8.00*E* − 10	1.05*E* − 03	5.01*E* − 06
0.2	1.85*E* − 06	1.21*E* − 09	1.19*E* − 09	4.88*E* − 09	1.19*E* − 09	1.19*E* − 09	1.19*E* − 09	8.05*E* − 04	1.73*E* − 06
0.3	3.20*E* − 05	9.34*E* − 09	5.85*E* − 09	2.22*E* − 07	6.96*E* − 09	5.62*E* − 09	5.61*E* − 09	6.71*E* − 04	8.38*E* − 07
0.4	2.45*E* − 04	1.44*E* − 07	2.11*E* − 08	3.94*E* − 06	6.87*E* − 08	1.12*E* − 08	1.04*E* − 08	6.39*E* − 04	9.98*E* − 07
0.5	1.20*E* − 03	2.23*E* − 06	2.30*E* − 07	3.79*E* − 05	1.12*E* − 06	5.31*E* − 08	2.34*E* − 08	6.79*E* − 04	1.67*E* − 06
0.6	4.50*E* − 03	2.23*E* − 05	2.47*E* − 06	2.45*E* − 04	1.24*E* − 05	6.38*E* − 07	7.10*E* − 08	7.72*E* − 04	5.17*E* − 06
0.7	1.40*E* − 02	1.62*E* − 04	2.03*E* − 05	1.21*E* − 03	9.75*E* − 05	7.55*E* − 06	6.33*E* − 07	9.10*E* − 04	4.77*E* − 06
0.8	3.84*E* − 02	9.20*E* − 04	1.34*E* − 04	4.97*E* − 03	5.98*E* − 04	6.89*E* − 05	7.82*E* − 06	1.07*E* − 03	9.40*E* − 06
0.9	9.63*E* − 02	4.39*E* − 03	7.46*E* − 04	1.78*E* − 02	3.05*E* − 03	5.02*E* − 04	8.06*E* − 05	1.29*E* − 03	6.62*E* − 06
1.0	2.27*E* − 01	1.84*E* − 02	3.71*E* − 03	5.74*E* − 02	1.36*E* − 02	3.07*E* − 03	6.80*E* − 04	1.98*E* − 03	7.58*E* − 05

Mean	3.82*E* − 02	2.39*E* − 03	4.61*E* − 04	8.16*E* − 03	1.73*E* − 03	3.65*E* − 04	7.69*E* − 05	9.86*E* − 04	1.12*E* − 05

**Table 5:** Statistical analysis of results based on values of absolute error.

*t*	PSO				PSO-ASA			
	MIN	MAX	MEAN	STD	MIN	MAX	MEAN	STD
0.1	1.0506*E* − 03	2.2340*E* − 01	9.2274*E* − 02	5.1432*E* − 02	3.1813*E* − 08	2.1601*E* − 03	9.6880*E* − 05	2.7723*E* − 04
0.2	8.0518*E* − 04	2.4845*E* − 01	1.0033*E* − 01	5.5966*E* − 02	3.4372*E* − 08	2.2102*E* − 03	9.7567*E* − 05	2.7860*E* − 04
0.3	6.7109*E* − 04	2.7628*E* − 01	1.1029*E* − 01	6.2147*E* − 02	8.3812*E* − 07	2.1940*E* − 03	1.0290*E* − 04	2.8024*E* − 04
0.4	6.3908*E* − 04	3.2281*E* − 01	1.2356*E* − 01	7.0441*E* − 02	1.2269*E* − 07	2.3203*E* − 03	1.1739*E* − 04	3.0450*E* − 04
0.5	6.7853*E* − 04	4.2304*E* − 01	1.4202*E* − 01	8.1644*E* − 02	3.0549*E* − 07	2.7264*E* − 03	1.3725*E* − 04	3.5327*E* − 04
0.6	7.7246*E* − 04	5.4096*E* − 01	1.6843*E* − 01	9.7069*E* − 02	9.4072*E* − 07	3.3844*E* − 03	1.6471*E* − 04	4.2624*E* − 04
0.7	9.0997*E* − 04	6.8783*E* − 01	2.0732*E* − 01	1.1879*E* − 01	4.7794*E* − 08	4.2143*E* − 03	2.1027*E* − 04	5.3421*E* − 04
0.8	1.0663*E* − 03	8.8150*E* − 01	2.6607*E* − 01	1.5005*E* − 01	5.0503*E* − 07	5.4046*E* − 03	2.7398*E* − 04	6.8757*E* − 04
0.9	1.2908*E* − 03	1.1516*E* + 00	3.5688*E* − 01	1.9625*E* − 01	4.0723*E* − 06	7.5318*E* − 03	3.8273*E* − 04	9.5844*E* − 04
1.0	1.9755*E* − 03	1.5506*E* + 00	5.0247*E* − 01	2.6814*E* − 01	2.9828*E* − 06	1.0874*E* − 02	5.6390*E* − 04	1.3640*E* − 03

**Table 6:** Comparative analysis of the results.

Parameters	*h* = 0.1		*h* = 0.05	
	PSO	PSO-ASA	PSO	PSO-ASA
GMAE	1.9576*E* − 01	2.0372*E* − 04	1.8444*E* − 01	6.3211*E* − 04
MF	1.1837*E* − 02	4.6577*E* − 06	1.0804*E* − 02	7.1294*E* − 06
MAE ≤ 10^-02^	21%	100%	22%	100%
MAE ≤ 10^-03^	01%	100%	00%	099%
MAE ≤ 10^-04^	00%	095%	00%	083%
FA ≤ 10^-03^	50%	100%	55%	100%
FA ≤ 10^-04^	05%	100%	05%	100%
FA ≤ 10^-05^	01%	099%	00%	098%
MIN-ET	075.23 s	079.29 s	132.42 s	157.14 s
MAX-ET	103.15 s	113.74 s	145.67 s	191.32 s
M-ET	076.93 s	092.17 s	142.43 s	183.57 s
STD-ET	002.93 s	007.32 s	002.02 s	007.62 s
